# Anti-Diabetic Effects of Oleuropein

**DOI:** 10.3390/metabo14110581

**Published:** 2024-10-27

**Authors:** Michael Iantomasi, Matthew Terzo, Evangelia Tsiani

**Affiliations:** 1 Department of Health Sciences, Brock University, St. Catharines, ON L2S 3A1, Canada; 2 Centre for Bone and Muscle Health, Brock University, St. Catharines, ON L2S 3A1, Canada

**Keywords:** type 2 diabetes mellitus, insulin resistance, polyphenols, oleuropein, anti-inflammatory, anti-diabetic

## Abstract

**Background/Objectives**: Oleuropein, a secoiridoid polyphenol found in olive oil as well as the fruit and leaves of the olive tree, has been reported to have antioxidant, cardioprotective, anti-inflammatory, anti-cancer, and anti-diabetic properties. Type 2 diabetes mellitus (TD2M) is a chronic metabolic disease characterized by impaired insulin action, termed insulin resistance. The development of T2DM is closely associated with obesity and chronic low-grade inflammation. In recent years, a rise in sedentary lifestyles and diets rich in refined carbohydrates and saturated fats has contributed to an increase in the prevalence of obesity and TD2M. Currently, the strategies for treating T2DM and its prevention lack efficacy and are associated with adverse side effects. Hence, there is an urgent need for novel treatment strategies, including naturally occurring compounds possessing hypoglycemic and insulin-sensitizing properties. **Methods**: This review summarizes the evidence of the anti-inflammatory and anti-diabetic properties of oleuropein from in vitro and in vivo animal studies, as well as the available clinical trials. **Results**: The existing evidence indicates that oleuropein may exert its anti-inflammatory effects by downregulating the levels of pro-inflammatory cytokines in hepatic and adipose tissue. Additionally, the evidence suggests that oleuropein targets skeletal muscle and enhances glucose uptake and its related protein signalling cascades, improving glucose tolerance and insulin sensitivity. **Conclusions**: Despite the evidence of oleuropein’s anti-inflammatory and anti-diabetic potential, more animal and clinical studies are needed to proceed towards its clinical/therapeutic use for metabolic diseases confidently.

## 1. Introduction

### 1.1. The Role of Insulin in Glucose Homeostasis and Insulin Action

Insulin is a central regulatory protein hormone secreted from the β pancreatic cells in the islets of Langerhans. Insulin is pivotal in the overall metabolism of carbohydrates, lipids, and proteins [1,2,3,4,5]. Insulin’s primary target tissues express the insulin receptor, a receptor tyrosine kinase [6]. Postprandially, the spike in blood glucose levels is detected by the pancreatic β cells, resulting in insulin secretion. Insulin, through circulation, reaches and acts on its primary target tissues, skeletal muscle, adipose, and liver. Insulin action in these tissues is initiated by binding of insulin to its receptor, leading to its activation and phosphorylation of tyrosine residues on the insulin receptor substrate (IRS), resulting in activation of the phosphoinositide 3 kinase (PI3K) protein kinase B (PKB/Akt) signalling cascade [6]. In muscle and adipose tissues, insulin increases glucose uptake, while in the liver, it inhibits glycogenolysis and gluconeogenesis, reducing endogenous (hepatocyte) glucose production [6]. Overall, the action of insulin in its target tissues brings the postprandially elevated blood glucose levels back to normal. Insulin therefore plays an important role in glucose homeostasis, and impairment in insulin action, termed insulin resistance, leads to glucose intolerance and the development of metabolic dysfunction.

### 1.2. Type 2 Diabetes Mellitus and Insulin Resistance

Type 2 diabetes mellitus (T2DM) has emerged as a major public health threat worldwide. It is estimated that the global prevalence of diabetes will rise from 9.3% (463 million people) in 2019 to 10.2% (578 million) by 2030 and 10.9% (700 million) by 2045 [7]. As mentioned, insulin resistance leads to metabolic dysfunction and T2DM. Complications of T2DM range from serious to life-threatening, including dyslipidemia, neuropathy, nephropathy, retinopathy, ketoacidosis, and others [8]. Clinical management of T2DM and its associated comorbidities is challenging, and the disease is associated with an increased rate of mortality. Systemic insulin resistance has long been associated with overnutrition and increased dietary consumption of refined carbohydrates and saturated fat [5,8]. This excessive intake of carbohydrates and lipids leads to adipocyte hypertrophy and the expansion of adipose tissue, leading to obesity [5,8]. In obese individuals with a body mass index (BMI) of 30 kg/m^2^ or greater [9], increased release of fatty acids from adipose tissue contributes to elevated levels of circulating fatty acids [8,10]. Intracellular lipid metabolites of fatty acids, diacylglycerols (DAGs), may impair insulin action through inflammatory pathways, such as novel isoforms of protein kinase C (PKC), c-Jun-N-terminal kinase (JNK), and inhibitor of κB kinase (IKK), which have been identified to phosphorylate IRS on serine residues and decrease insulin sensitivity [11,12]. Additionally, recent evidence indicates that obesity and consumption of a high-fat diet (HFD) alter the permeability of the intestinal walls, termed leaky gut, which contributes to chronic low-grade inflammation through activation of the innate immune system, leading to the development of insulin resistance and T2DM [13].

### 1.3. The Role of Phytochemicals in Modern Medicine and Drug Development

In recent years, the scientific community has focused on finding plant-derived chemicals with anti-obesity and anti-diabetic properties. Historically, plant-derived chemicals have been widely used in the development of drugs to treat various diseases. Metformin, a biguanide, was originally isolated from the French lilac (*Galega officinalis*) [14], and has since been developed as the gold standard medication for T2DM, acting as a hypoglycemic agent that effectively lowers blood glucose levels. Currently, many different plant extracts and phytochemicals, such as resveratrol, curcumin, quercetin, berberine, carnosol, ursolic acid, chlorogenic acid, capsaicin, piperine, and lycopene, are widely available and can be purchased as natural supplements. However, these compounds do not have FDA approval and are not used in clinical practice as prescription medication. More organized animal and clinical studies are necessary before recommendations for use in the general public are made. More future clinical studies are required to fully understand their impact on human health.

### 1.4. The Structure, Metabolism and Bioavailability of Oleuropein

**Figure 1:**
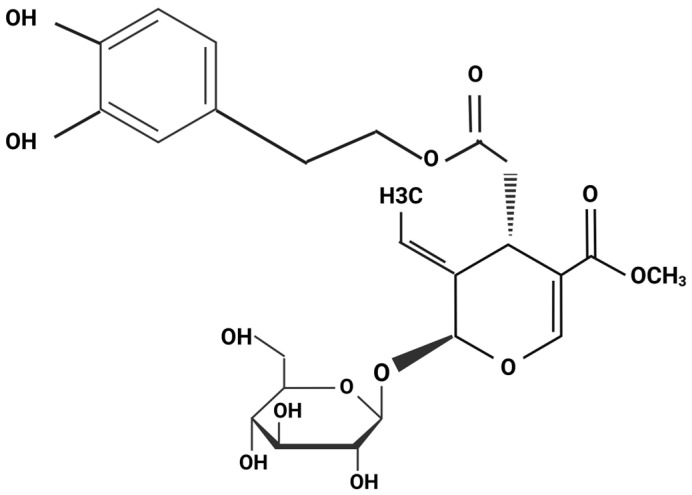
Chemical structure of oleuropein (OLE).

The common use of olives and olive oil in Mediterranean cooking has drawn attention to the therapeutic potential of olive and olive leaf polyphenol compounds. Oleuropein (OLE), tyrosol, and hydroxytyrosol (Htyr) are the most abundant polyphenols in olive leaf extracts [15]. OLE is highly concentrated in the leaves (60–90 mg/g) and fruit (140 mg/g) of the olive tree *Olea europaea* L. [16]. The focus of this review is on OLE, seen in Figure 1, a phytochemical that belongs to the subgroup of polyphenols, secoiridoids. Following oral administration, OLE remains relatively stable in the stomach but undergoes extensive metabolism in the large intestine, where it undergoes deglycosylation, giving rise to oleuropein aglycone (OLE aglycone) [17]. OLE aglycone may be further metabolized into Htyr by de-esterification [17]. Oleuropein has been reported to have antioxidant [18], cardioprotective [19], anti-inflammatory [20], anti-cancer [21], and anti-diabetic [15] properties.

In the present review, we summarize the current evidence of the anti-diabetic and anti-inflammatory properties of OLE. We reviewed in vitro and in vivo studies utilizing models of insulin resistance and T2DM. To gather this evidence, PubMed, Google Scholar, Dimensions, and Scopus searches were conducted using the following keywords: “oleuropein and muscle and insulin resistance”, “oleuropein and adipocytes/adipose tissue”, “oleuropein and hepatocytes”, “oleuropein and beta-pancreatic cells”, and “oleuropein and macrophages”. All relevant studies are presented in chronological order, both in text and in table format. Articles were excluded if they were not specific to the topic or were not available in English.

The majority of studies summarized in this manuscript have been peer-reviewed, having undergone method validation and data integrity checks during the review process. We examined the methodology sections of all the original published studies, further confirming the validity of the bioassay methods used. Although we lacked access to raw data, a close examination of the reported statistical analyses in the original studies indicated their validity. The average sample sizes in the original studies were within acceptable ranges, 3–6 for in vitro and 5–8 for in vivo studies. Significance was predominantly set at *p* < 0.05, with many studies employing more stringent levels of *p* < 0.01 and *p* < 0.001.

In our summarized text and tables, we report statistically significant findings using the terms “increase” or “decrease”, reflecting statistically significant differences reported in the original research articles. We have only included changes/data that were reported as statistically significant in the primary studies. Readers interested in specific statistical parameters such as the confidence intervals, effect sizes, or statistical power of individual studies are encouraged to consult the original research articles, where such detailed information may be available.

## 2. Anti-Diabetic Effects of Oleuropein: In Vitro Studies

### 2.1. Effects of Oleuropein on Skeletal Muscle Cells

Hadrich et al. [22] exposed C2C12 muscle cells to OLE and found increased glucose consumption and increased protein levels of phosphorylated/activated AMPK, ACC, and ERK [22]. In addition, OLE significantly decreased H_2_O_2_-induced reactive oxygen species (ROS) in comparison to untreated cells, thereby demonstrating antioxidant properties (Table 1) [22].

Fujiwara et al. [23] exposed C2C12 myotubes to OLE and found a significant increase in glucose uptake, comparable to the effects of insulin. In contrast to insulin, OLE did not increase protein content of phosphorylated/activated Akt; however, it did increase protein levels of phosphorylated/activated AMPK [23], in agreement with the above study by Hadrich et al. [22]. In addition, pretreatment with OLE increased GLUT4 mRNA levels and restored insulin-stimulated glucose uptake in the presence of palmitate, through an attenuation in palmitate-induced decreases of phosphorylated/activated AMPK (Table 1) [23].

Kikusato et al. [24] exposed cultured avian muscle cells to OLE and found increased mitochondrial biogenesis, increased avian uncoupling protein (avUCP), PGC1-α, TFAM, NRF1, ATP5a1, and SIRT1 mRNA levels and cytochrome c oxidase activity, and decreased mitochondrial superoxide activity significantly indicating a reduction in ROS production.

Exposure of isolated rat soleus muscle to OLE increased phosphorylated/activated AMPK, GLUT4 protein content, and glucose uptake [25]. The use of compound C, an AMPK inhibitor, abolished these effects, while wortmannin, a PI3K inhibitor, did not affect the OLE-induced effects. Collectively, these findings demonstrate the potential of OLE to promote GLUT4-mediated glucose uptake in muscle via an AMPK-dependent mechanism [25].

Alkhateeb et al. [26] found that treatment of soleus muscle isolated from male Sprague–Dawley rats with OLE attenuated palmitate-induced insulin resistance and improved phosphorylation/activation of AS160, GLUT4 translocation, and insulin-stimulated glucose uptake. Additionally, OLE treatment increased phosphorylation/activation of AMPK, and the use of compound C, an AMPK inhibitor, abolished these effects of OLE. Collectively, these findings demonstrate the potential of OLE to attenuate palmitate-induced insulin resistance in muscle by activating the AMPK/AS160/GLUT4 pathway.

### 2.2. Effects of Oleuropein on Adipocytes

Drira et al. [27] treated 3T3-L1 mouse adipocytes with OLE and reported a marked decrease in adipocyte differentiation and total lipid content, without affecting cell viability [27]. OLE further reduced GDPH activity, a marker of triglyceride synthesis, and total triglyceride levels [27]. These effects were associated with downregulation of mRNA levels of pro-lipogenic genes sterol regulatory element binding protein 1c (SREBP-1c) and fatty acid synthase (FASN) [27]. Dose-dependent downregulation of mRNA levels of PPARγ and C/EBPα, along with some of their target genes, CD36 and GLUT4, was seen following OLE treatment [27]. Decreases in the aforementioned target genes were associated with a decrease in glucose and fatty acid uptake by adipocytes [27]. OLE attenuated adipocyte clonal expansion through impediment of the cell cycle, with an increase in adipocytes in G0/G1 and S phases (Table 2) [27].

Svobodova et al. [28] treated 3T3-L1 adipocytes with OLE and found a significant attenuation of adipocyte differentiation coupled with a downregulation in mRNA levels of adipogenic genes: PPARγ, C/EBPα, SREBP-1c, and FASN (Table 2) [28].

Kuem et al. [29] treated 3T3-L1 preadipocytes with OLE and found a dose-dependent reduction in adipocyte differentiation, indicating anti-adipogenic effects [29]. Incubation with OLE resulted in a significant attenuation of intracellular lipid aggregation [29]. OLE protected against secreted frizzled-related protein 2 (SFRP2) and galnon-induced lipid accumulation [29]. Similarly, OLE reduced mRNA levels of PPARγ, C/EBPα, and their target genes fatty acid synthase (FASN) and lipoprotein lipase (LPL), demonstrating the polyphenol’s anti-adipogenic effects (Table 2) [29].

Hadrich et al. [30] examined the metabolic effects of OLE in 3T3-L1 adipocytes and found no alteration in protein levels of p-Akt or PI3Kp85 [30] (Table 2).

### 2.3. Effects of Oleuropein on Hepatocytes

Hur et al. [31] treated human hepatoma-derived (HepG2) cells and mouse hepatocyte cells (FL38B) with OLE and found an attenuation of the FFA-induced lipid accumulation, indicating effects against hepatic steatosis [31]. Furthermore, OLE (10 µM) significantly decreased lipid droplet size in both HepG2 and FL38B cells [31]. The OLE treatment prevented FFA-induced increases in protein levels of phosphorylated/activated ERK [31]. OLE treatment had no effect on mRNA levels of adipose differentiation-related protein (ADRP), a lipid droplet-associated protein, or mRNA levels of tail interacting protein of 47 kDa (TIP47) [31]. Furthermore, OLE had no effect on protein levels of phosphorylated/activated Akt or JNK (Table 3) [31].

Vergani et al. [32] exposed FaO rat hepatoma cells to OLE and found a significant reduction in cellular triglyceride content, indicating attenuation of FFA-induced steatosis [32]. In addition, OLE reduced the FFA-induced increase in malondialdehyde (MDA) levels, a marker of lipid peroxidation (Table 3) [32].

Malliou et al. [33] performed molecular docking simulations, which revealed that in its integral form, OLE is structurally compatible with the PPARα binding cavity [33]. Likewise, a luciferase reporter gene assay revealed an increase in PPRE-luc plasmid activity indicating an ability to activate PPARα and leading to subsequent heterodimerization with RXR [33]. Furthermore, treatment of HepG2 cells with OLE increased mRNA and protein levels of PPARα and mRNA levels of several downstream PPARα target genes: aCYL-CoA oxidase 1 (ACOX1), cytochrome P450 family 4 subfamily 3 polypeptide 14 (CYP4A14), lipin 1, and acyl-CoA thioesterase 4 (ACOT4) (Table 3) [33].

Santini et al. [34], using HepG2 cells, found a significant reduction in palmitate-induced hepatic lipid accumulation with OLE treatment.

Treatment of alpha mouse liver cells (AML-12) with an OLE-rich extract containing ~69% OLE attenuated the oleic acid-induced TG accumulation that was associated with reduced mRNA levels of pro-lipogenic genes ACC, SREBP1-c, and FASN [35]. These data suggest OLE has the ability to attenuate fatty acid-induced lipid accumulation and, subsequently, hepatic steatosis in liver cells. Moreover, OLE treatment abrogated oleic acid-induced reductions in mRNA levels of LKB1, PGC-1α, Tfam, and Nrf2, with no effect on total AMPK levels (Table 3) [35].

**Figure 2:**
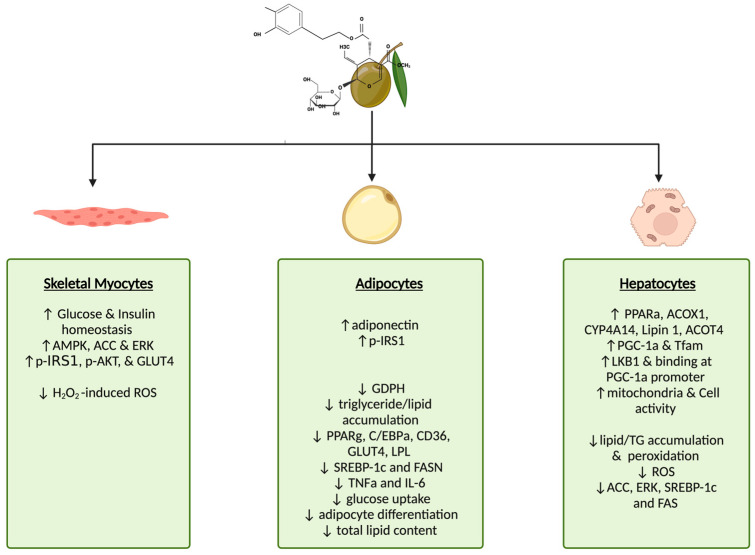
Effects of oleuropein on insulin target tissues, skeletal myocytes, adipocytes, and hepatocytes in vitro. The above figure created using BioRender (https://BioRender.com/c29m951) is based on the data from the cited studies [22,23,27,28,29,30,31,32,33,35].

Overall, the evidence summarized above indicates the effects of oleuropein’s ability to increase skeletal muscle glucose uptake, attenuate FFA-induced insulin resistance, and decrease ROS levels. Furthermore, in adipocytes, oleuropein decreased fatty acid and glucose uptake, abrogated lipid accumulation, and attenuated increases in pro-inflammatory signalling. In hepatocytes, oleuropein increased mitochondrial activity while decreasing lipid accumulation and the induction of adipogenic genes (Figure 2).

### 2.4. Effects of Oleuropein on Pancreatic Beta Cells

Cumaoğlu et al. [36] examined the effects of OLE on rat insulinoma cells (INS-1) and reported an attenuation of H_2_O_2_-mediated decline in cell viability and H_2_O_2_-induced ROS production [36]. Importantly, OLE attenuated the H_2_O_2_-induced reduction in insulin secretion (Table 4) [36].

Cumaoğlu et al. [37] treated INS-1 cells with OLE and witnessed an attenuation of cytokine (TNF-α, IL-1β, and IFN-γ)-induced decline in cell viability, with the abolition of the cytokine-mediated increase in ROS levels [37]. Similarly, OLE protected against cytokine-induced alterations in superoxide dismutase (SOD), indicating its antioxidant properties [37]. OLE was able to significantly reverse cytokine-induced decreases in insulin secretion. Overall, these data provide evidence of the protective effects of OLE against cytokine/ROS-induced defects in β pancreatic cells and the restoration of their capacity to release insulin.

Treatment of INS-1 cells with OLE was found to increase total insulin content without significantly altering glucose-stimulated insulin release (Table 4) [38].

Choi et al. [39] examined the effects of OLE on 2,3,7,8-tetrachlorodibenzo-p-dioxin (TCDD)-induced beta cell damage, utilizing INS-1 cells. TCDD, an organic pollutant, contributes to diabetes in humans through an induction of beta cell stress and its related metabolic effects [39]. OLE attenuated TCDD-induced increases in mRNA levels of prostaglandin E2 (PGE_2_), COX-1, and phospholipase A_2_ (iPLA_2_) [39]. Furthermore, OLE reversed TCDD-induced increases in cytosolic mRNA levels of JNK, TNF-α, and ROS production. Lastly, OLE abolished the TCDD-induced decrease in mRNA levels of phosphorylated Akt and GLUT2 (Table 4) [39].

**Figure 3:**
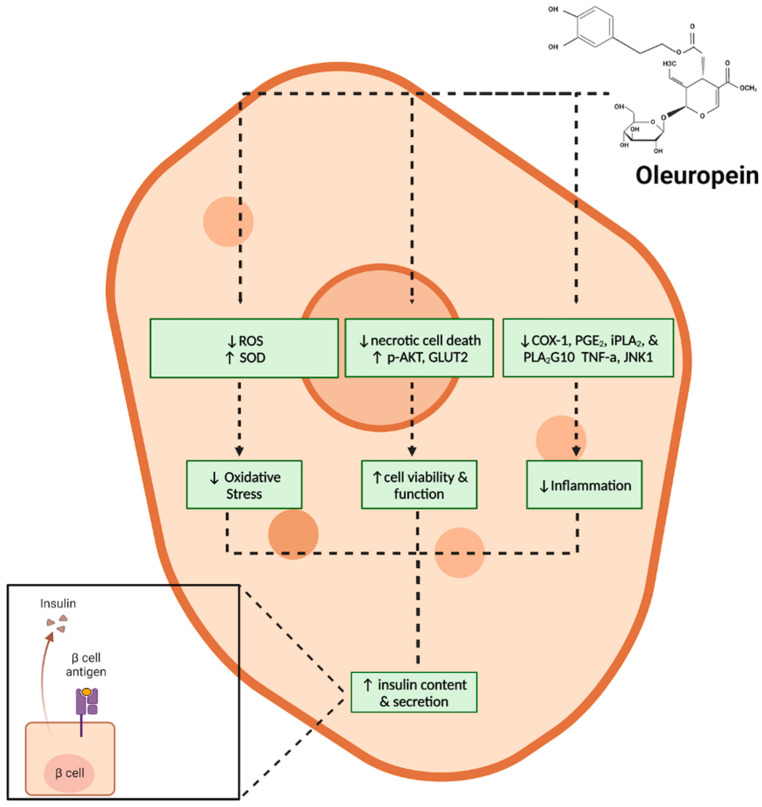
Effects of oleuropein on beta pancreatic cells. The above figure, created using BioRender (https://BioRender.com/c29m951), is based on the data of the cited studies [36,37,38,39].

In summation of the above discussion, oleuropein improves insulin content and secretion and decreases ROS production and levels of pro-inflammatory mediators (Figure 3).

## 3. Anti-Diabetic Effects of Oleuropein: In Vivo Animal Studies

### 3.1. Effects of Oleuropein on Diet-Induced Diabetes Animal Models

The ability of OLE to protect against the negative effects of a high-fat or “Western-style” diet (HFD) has been examined in several studies. Oi-Kano et al. [40] found that OLE could protect against HFD-induced weight gain in male Sprague–Dawley rats, indicating anti-obesogenic properties [40]. OLE administration led to significant reductions in HFD-induced increases in plasma TG, FFA, TC, and leptin levels [40]. Levels of UCP-1, a protein involved in adipocyte thermogenesis, were increased in the interscapular brown adipose tissue (iBAT) of rats supplemented with OLE [40] (Table 5) [40].

In a separate study, Jemai et al. [41] examined OLE’s effects on male Wistar rats fed a high-cholesterol diet (HCD) and found it protected against HCD-induced increases in the liver/bodyweight ratio [41]. Serum lipid analysis revealed OLE significantly attenuated HCD-induced increases in plasma levels of TC, TG, and LDL-C, [41]. Furthermore, the phenolic compound was able to restore levels of HDL-C that had been previously reduced in HCD rats, indicating this compound’s hypolipidemic properties [41]. OLE treatment reversed the negative effects of an HCD on hepatic antioxidant enzymes SOD and CAT [41]. OLE supplementation increased anti-oxidant activity as indicated by restoration of ABTS scavenging ability and decreased lipid peroxidation in the liver, heart, kidneys, and aorta of HCD-fed rats supplemented with the secoiridoid [41]. Treatment with OLE prevented the development of HCD-induced cardiac muscle hypertrophy and lesioning in the aortic wall [41]. Similarly, OLE prevented a shift of hepatocyte nuclei to the periphery and fatty cyst development (Table 5) [41].

Kim et al. [42] administered OLE (0.03% (*w*/*w*) in diet) to HFD-fed mice and found more than a two-fold difference in hepatic gene expression of over 90 genes, according to microarray analysis. Specifically, OLE treatment led to a reduction in hepatic genes involved in oxidative stress and inflammatory response. In addition, supplementation with OLE reduced mRNA levels of genes involved in the process of hepatic fatty acid uptake.

Park et al. [43] administered OLE (0.03% *w*/*w*) ad libitum for 10 weeks in mice and found a decrease in HFD-induced body and liver weight gain. OLE treatment led to an attenuation in elevated plasma AST and ALT as well as plasma and liver FFA, TC, and TG. Moreover, OLE significantly decreased liver mRNA levels of lipid regulating genes liver X receptor (LXR), fatty acid binding protein 2 (aP2), lipoprotein lipase (LPL), and PPAR*γ*2, as well as mRNA levels of cyclin D (Cyc-D), E2F transcription factor1 (E2F1), cathepsin S (CTSS), secreted frizzled-related protein 5 (SFRP5), and dickkopf homolog 2 (DKK2). Additionally, OLE treatment attenuated HFD-induced elevation in phosphorylated/activated ERK and a decrease in β-catenin levels. OLE attenuated the HFD-induced increase in mRNA levels of TLR2/4, myeloid differentiation factor 88 (MyD88), IL-1β, il-6, TNFα, interferon beta (IFNβ), TNF receptor superfamily member 6 (FAS), and TNF-related apoptosis-inducing ligand, indicating anti-inflammatory properties.

Kuem et al. [29] administered OLE to HFD-fed male mice and witnessed a significant reduction in bodyweight gain coupled with a decrease in total visceral fat-pad mass, accomplished through a decrease in adipocyte size. In conjunction with the previous findings, OLE attenuated HFD-induced increases in serum TC, TG, and FFA levels. In epidydimal adipose tissue, OLE decreased mRNA levels of SFRP2 and dickkopf 2 (DKK2) while increasing mRNA levels of Wnt10b. Similarly, OLE decreased mRNA levels of galanin, galanin receptor 1 (GalR1), galnin receptor 2 (GalR2), PKCδ, and resistance to audiogenic seizures (RAS). Treatment with OLE led to elevated protein levels of β-catenin while decreasing phosphorylated/activated ERK. Furthermore, OLE decreased the mRNA levels of adipogenic genes PPAR*γ*, C/EBPα, LPL, FASN, and adaptor complex protein 2 (aP2) (Table 5).

Lepore et al. [44] investigated the protective effects of oleuropein (OLE) in high-fat, “cafeteria diet”-fed C57BL/6J mice [44]. OLE (or its peracetylated derivative (AC-OLE) was supplemented in drinking water for a 15-week period [44]. Significant increases in body weight, abdominal growth, and abdominal fat as a result of increased energy intake, with no differences in food intake, were seen with HFD feeding [44]. Contrary to this, supplementation with OLE or its derivative AC-OLE protected against these effects [44]. Furthermore, OLE and AC-OLE seemed to protect against HFD-induced liver enlargement and hepatic steatosis, which was evident through histological analysis and reductions in liver weight in mice supplemented with either compound [44]. Supplementation with OLE or AC-OLE was able to protect against HFD-induced increases in plasma levels of LDL, alanine transaminase (ALT), and aspartate transaminase (AST). However OLE was not able to decrease AST levels to an extent comparable to the standard diet [44]. OLE and its derivative were able to attenuate HFD-induced increases in blood glucose, insulin, and leptin levels as well as HOMA-IR (Table 5) [44].

Hadrich et al. [45] examined the effects of 8-week OLE supplementation in high-cholesterol-fed male Wistar rats. While high-cholesterol feeding led to marked increases in white adipose tissue, body weight, and adipocytes in epididymal adipose tissue, OLE supplementation attenuated these HCD-induced effects [45]. Moreover, OLE demonstrated its hypocholesterolemic properties through abolition of HCD-induced increases in serum levels of ALT, triglycerides, total cholesterol, and LDL, while significantly increasing levels of HDL-C and adiponectin mRNA [45]. OLE was further able to protect against HCD-induced increases in liver weight and fat deposition, indicating its anti-steaotic effects [45]. In adipose tissue, OLE supplementation abolished HCD-induced increases in protein levels of adipogenic markers PPARγ, C/EBPα, and FAS while increasing the level of phosphorylated/activated AMPK (Table 5) [45].

Ramadan et al. [46] administered OLE (10 mg/kg/day) to male albino rats and found that OLE attenuated the HFD-induced increase in total body weight, serum total cholesterol, and LDL cholesterol, while a significant increase in serum HDL cholesterol was observed. Additionally, OLE administration resulted in a significant reduction in serum IL-6. That study demonstrated the strong potential of OLE to protect against HFD-induced weight gain, lipid dysregulation, and inflammation.

Fujiwara et al. [23] examined OLE supplementation in HFD mice and found no effect on white or brown adipose tissue weight. Similarly, OLE had no effect on fasting blood insulin, AUC, or blood levels of non-esterified fatty acids. Meanwhile, OLE decreased fasting blood glucose levels and HOMA-IR. Following OLE supplementation, an increase in GLUT4 levels in muscle tissue was seen through the quantification of fluorescence immunohistochemistry (Table 5).

Santini et al. [34] administered OLE (0.03% *w*/*w*) for 8 weeks to HFD-fed male and female mice and found reductions in heart weight and serum AST, ALT, and TC in both genders. Similarly, in both genders, OLE increased serum HDL-C levels while decreasing serum levels of pro-inflammatory marker G-CSF. OLE attenuated elevations in serum IL-1α in males and IL-2 in female mice. In both genders, administration of OLE decreased hepatic protein levels of CAT, SIRT1, and SIRT3 while decreasing SOD levels only in female mice. Additionally, OLE decreased hepatic MDA levels in both genders. Collectively, these findings indicate OLE’s ability to protect against HFD-induced hepatic steatosis and improve serum lipid profiles.

Hadrich et al. [30] treated high-fat-diet-fed male rats with OLE and found no effect on bodyweight gain. However, OLE supplementation did decrease blood glucose and insulin levels as well as AUC and HOMA-IR indexes. Similarly, treatment with OLE led to a reduction in serum TG, TC, and LDL-C while increasing the serum level of HDL-C. Immunohistochemistry (IHC) revealed that OLE attenuated HFD-induced decreases in liver and WAT p-Akt, GLUT4, and pancreatic IRS1 levels. IHC analysis further revealed that OLE decreased levels of TNFα in the liver and WAT (Table 5).

Hou et al. [35] treated HFD-fed mice with OLE and found significant decreases in bodyweight gain and liver weight combined with decreased hepatocyte swelling and ballooning. Moreover, OLE supplementation decreased serum levels of ALT, AST, TC, TG, and LDL-C. Similarly, OLE treatment decreased mRNA levels of pro-inflammatory factors MCP-1 and CD68 while also decreasing mRNA levels of adipogenic markers SREBP1c, ACC, and FASN. In turn, OLE increased mRNA levels of PGC-1α, Tfam, Nrf2, mitochondrial DNA (mtDNA), and UCP2 (Table 5).

Zhou and Dou [47] supplemented HFD-fed mice with OLE (0.03% *w*/*w*) for 16 weeks and found attenuation in body weight gain, epididymal fat mass gain, and hepatic lipid accumulation. OLE administration prevented HFD-induced elevations in serum AST, ALT, TC, TG, and LDL-C. OLE reduced hepatic MDA, GPx, SOD, and CAT levels, demonstrating antioxidant properties. Liver metabolomic analysis revealed that OLE increased levels of liver metabolites including nicotinamide, tauroursodeoxycholic acid, taurine, and docosahexaenoic acid. OLE treatment resulted in elevations in hepatic fatty acid oxidation markers PPARα, CPT1α, and acyl-coenzyme A oxidase 1 (ACOX1) while reducing protein levels of pro-lipogenic proteins FAS, SREBP-1c, and SCD1. Moreover, OLE supplementation led to reductions in hepatic mRNA levels of pro-inflammatory markers IL-6, TNFα and MCP-1.

Schirone et al. [48] treated HFD-fed mice with OLE (1.8 mg/kg) for 8 weeks and found significant reductions in liver steatosis and fibrosis. OLE attenuated increases in hepatocyte LPS localization as well as increased levels of TLR4+ macrophages and CD42b+ platelets, indicating anti-inflammatory properties. Additionally, OLE decreased serum levels of LPS, zonulin, sP-selectin, high-density lipoprotein 3 (HDL3), TNFα, and IFN*γ*. In the intestine, OLE decreased goblet cell presence and crypt length. Moreover, OLE administration led to reductions in enterocyte LPS localization and occludin abundance coupled with a decrease in the number of TLR4+ macrophages. Collectively, these data indicate that OLE may protect against HFD-induced inflammation and hepatic steatosis.

### 3.2. Effects of Oleuropein on Alloxan-Induced Diabetes Animal Models

Al-Azzawie and Alhamdani [49] investigated the hypoglycemic and antioxidant effects of OLE supplementation in alloxan-induced diabetic rabbits over a 16-week period. Plasma and erythrocyte levels of MDA, a prominent marker of oxidative stress, were found to be increased in diabetic controls, whereas OLE supplementation protected against these effects [49]. Furthermore, OLE abolished alloxan-induced decreases in glutathione peroxidase (GPx), glutathione reductase (GRx), catalase (CAT), glutathione (GSH), α-tocopherol, β-carotene, and ascorbic acid, while decreasing levels of superoxide dismutase, indicating the compound’s anti-oxidant properties [49]. Likewise, OLE attenuated alloxan-induced increases in blood glucose levels (Table 6) [49].

Jemai et al. [50] examined the anti-diabetic and antioxidant effects of OLE in alloxan- induced diabetic adult male Wistar rats over a four-week period. Treatment of diabetic rats with OLE attenuated alloxan-induced increases in blood glucose while leading to increases in hepatic glycogen storage [50]. Similarly, total cholesterol levels that had been elevated in diabetic animals were reduced with OLE administration [50]. Alloxan-induced decreases in hepatic SOD levels and CAT activity were significantly restored following administration of OLE [50]. Similarly, OLE supplementation protected against alloxan-induced decreases in antioxidant properties, as indicated by an increase in TEAC values and a decrease in TBARS, a marker of lipid peroxidation [50]. Photomicrographic analysis of liver tissue samples revealed that OLE prevented against alloxan-induced hepatocyte vacuolation, peripheral nuclei, and fatty cyst presence, indicating the ability of the compound to protect against hepatic steatosis (Table 6) [50].

### 3.3. Effects of Oleuropein on Streptozotocin-Induced Diabetes Animal Models

Khalili et al. [51] investigated the effects of OLE on male Sprague–Dawley rats with simultaneous type 2 diabetes (nicotinamide and streptozotocin injection) and renovascular hypertension (left renal artery clip), over a four-week period [51]. The systolic blood pressure (SBP) of OLE-supplemented diabetic and hypertensive rats was significantly lower compared with untreated rats with similar conditions [51]. OLE administration also abrogated streptozotocin-induced increases in fasting blood glucose (FBG) levels [51]. Moreover, serum total cholesterol, LDL-C, and triglyceride levels were significantly elevated in diabetic and diabetic/hypertensive control rats, whereas OLE supplementation annulled these effects [51]. Furthermore, OLE protected against streptozotocin-induced decreases in serum HDL-C levels [51]. ELISA analysis revealed marked reductions in insulin levels in diabetic and diabetic/hypertensive rats, which were abrogated with OLE supplementation [51]. OLE was able to prevent streptozotocin-induced decreases in antioxidant activity, represented by decreases in serum MDA levels and an increase in erythrocyte SOD levels (Table 7) [51].

Mohamed et al. [52] orally administered OLE (5 mg/kg BW/day, for 15 days) to STZ-induced diabetic rats and found that OLE decreased ALT, AST, alkaline phosphatase, and bilirubin and increased G6PDH serum protein, suggesting improved hepatic function. Additionally, OLE supplementation decreased serum total cholesterol, LDL cholesterol, and triacylglycerol while increasing serum HDL cholesterol, indicating an improved lipid profile. OLE treatment reduced serum leptin and increased adiponectin protein levels, while liver TNF-α and COX-2 mRNA levels were reduced. OLE increased glutathione peroxidase, catalase, glutathione-S-transferase, and superoxide dismutase serum protein levels, all markers of antioxidant activity. Collectively, these findings suggest the potential of OLE to enhance hepatic function and attenuate diabetic-induced inflammation.

### 3.4. Effects of Oleuropein on Diabetes Phenotypical Animal Model

Zheng et al. [53] administered OLE orally to 17-week-old BKS-Lepr*^em2Cd479^*/Gpt (*db*/*db*) male mice for a 15-week period and found reduced plasma glucose levels and a reduced HOMA-IR index, indicating improved glucose tolerance and increased insulin sensitivity.

OLE treatment significantly reduced the percentage of epididymal fat [53]. Histological analysis revealed that OLE supplementation attenuated decreases in pancreatic islet area, hepatic lipid deposition, and the disorderliness of myocardial fibers due to the diabetic phenotype [53].

RT-PCR analysis revealed that OLE was able to significantly diminish the hepatic expression of PTP1B, a negative regulator of hepatic insulin signaling, while increasing protein levels of phosphorylated/activated Akt (Thr308) [53]. Furthermore, OLE decreased mRNA levels of fructose-1,6-biphosphatase-1 (Fbp1), a key enzyme in hepatic gluconeogenesis [53].

While the diabetic phenotype resulted in negative alterations in the gut, OLE supplementation reversed such negative effects [53]. Furthermore, treatment with OLE decreased the abundance of Gram-negative rod-shaped bacteria resulting from feeding with a high-fat diet. Spearman’s correlation analysis was performed to investigate associations between diabetes-related indexes and the related changes in intestinal flora between untreated and OLE-supplemented mice (Table 8). Treatment [54] of diabetic db/db mice with OLE (200 mg/kg a day) for 15 weeks resulted in reduced renal fibrosis and glomerular mesangial matrix expansion. In addition, OLE treatment alleviated inflammation and prevented the induction of apoptosis in renal and cardiac tissue. OLE reduced TUNEL-stained cells and decreased cleaved caspase-3 and BCL-associated X apoptosis regulator (BAX) immunohistochemical pathological sections, while B-cell lymphoma-2 (Bcl-2)-positive regions were increased. OLE administration attenuated increased mRNA levels of oxidase-related marker NADPH oxidase 4. Transcriptome analysis revealed that OLE treatment led to an upregulation in differentially expressed genes in renal tissue related to cGMP-PKG and Gap junction signaling, both considered to be favourable for improving the condition of diabetic nephropathy. Further transcriptome analysis of heart tissue indicated that OLE downregulated signaling pathways associated with p53 and cellular senescence, thereby counteracting associated side effects of diabetic cardiomyopathy.

**Figure 4:**
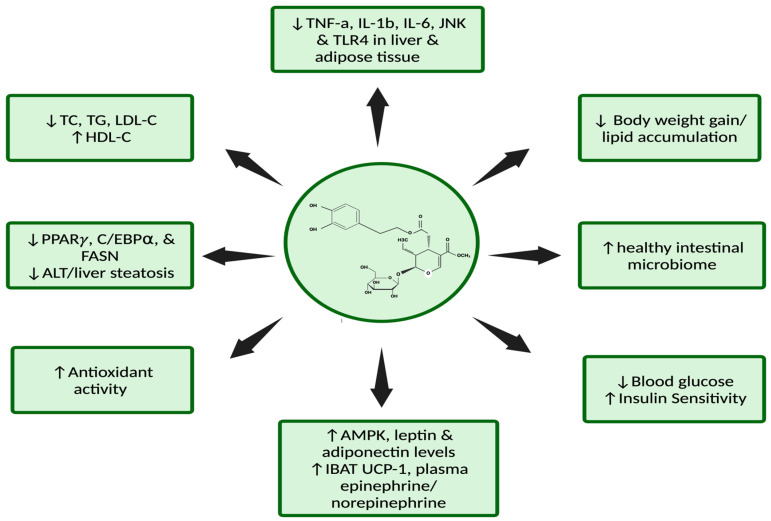
Summary of the anti-diabetic effects of oleuropein in in vivo animal models of diabetes. The above figure, created using BioRender (https://BioRender.com/c29m951), is based on the data of the cited studies [23,29,30,35,40,41,44,45,49,50,51,53].

In summary, in the in vivo studies described above, various doses of OLE (1 to 200 mg/kg) were administered, with the most common method being oral administration. The effects of OLE administration were investigated in various models of diabetes, including HFD, alloxan, STZ, and db/db in differing animals, including mice, rats, rabbits, and chicks. Despite the various models of induced diabetes and the utilized animals, the evidence indicates OLE attenuated total body and fat-specific mass gain while glucose tolerance and insulin sensitivity were improved. Additionally, OLE protected against the various models of diabetic-induced alterations of blood lipid profile, decreasing inflammatory signaling in both the liver and adipose tissue while preventing liver steatosis and increasing anti-oxidant activity (Figure 4).

## 4. Anti-Diabetic Effects of Oleuropein: Randomized Control Trial (RCT)

de Bock et al. [55] investigated the effects of olive leaf polyphenol supplementation in middle-aged overweight (BMI 25–30 kg/m^2^) men over a 12-week period [55]. Participants were administered four capsules containing over 80% oleuropein (51.12 mg per capsule or 204.49 mg daily) during the treatment period [55]. This supplementation led to an increase in daily energy intake (kcal) from sugars [55] and improvements in insulin sensitivity (IGTT) and glucose tolerance (OGTT). Furthermore, increased plasma concentrations of IGFBP-1/2 [55] and of the pro-inflammatory cytokine IL-6 (Table 9) [55] were seen. Although these data indicate the potential of this supplement (80% OLE) to improve insulin sensitivity and glucose tolerance in humans, it is unfortunately not clear whether the presence of other polyphenols (20%) and not OLE contributed to these effects. The increase in pro-inflammatory IL-6 levels is of concern and should be investigated in the future. Future studies utilizing pure OLE administration in humans should be performed to investigate the doses required, its anti-diabetic potential, and potential toxicity.

## 5. Anti-Diabetic Effects of Oleuropein: Macrophages

Ryu et al. [56] exposed RAW 264.7 cells to OLE, which resulted in a dose-dependent reduction in both LPS-induced iNOS mRNA levels and subsequent NO production, along with the pro-inflammatory marker, COX-2 [56]. Moreover, OLE was found to greatly reduce mRNA levels of other pro-inflammatory markers, IL-1β and IL-6 [56]. LPS-induced activation of the NFκB pathway was reduced on co-incubation with OLE, specifically through inhibition of phosphorylation of IκB-α, therefore inhibiting nuclear translocation of NFκBp65 [56]. OLE further reduced mRNA levels of ERK, JNK, and AP-1 (Table 10) [56].

Castejon et al. [57] incubated murine peritoneal macrophages with OLE and observed reductions in LPS-induced intracellular ROS production; however, little effect on the anti-oxidant proteins Nrf2 and HO-1 was noted [57]. LPS-induced NO production was attenuated through a reduction in iNOS protein levels [57]. OLE was further able to downregulate protein levels of COX-2 and mRNA levels of PGE_2_, two crucial factors regulating the arachidonic acid pathway [57]. LPS-induced increases in mRNA levels of IL-1β and IL-6 were significantly abrogated in the presence of OLE [57]. OLE resulted in dose-dependent reductions in protein levels of ERK, JNK, and p38, and this also held true for the reduction of STAT3, a pro-inflammatory transcription factor (Table 10) [57].

De Cicco et al. [58] pre-incubated RAW 264.7 macrophages with an olive leaf extract containing >75% OLE, which resulted in a significant, dose-dependent reduction in palmitate-induced increases in mRNA levels of TNFα, IL-6, and IL-1β, demonstrating the compound’s anti-inflammatory abilities. Additionally, OLE, in a dose-dependent manner, ameliorated palmitate-induced increases in ROS production [58]. Furthermore, OLE treatment led to upregulation in mRNA levels of anti-oxidant enzymes including glutamate–cysteine ligase catalytic unit (GCLC) and modulatory unit (GCLM), heme oxygenase-1 (HMOX-1), and SOD2 protein levels, demonstrating its antioxidant capabilities [58]. Moreover, pre-treatment with OLE decreased protein cytosolic levels of KEAP1 while, interestingly, increasing nuclear protein levels of NRF2 [58]. The protein content of M1 polarization markers iNOS and COX-2 as well as iNOS+ cells were increased following palmitate treatment; however, the presence of OLE attenuated such effects [58]. Similarly, palmitate-induced activation of NFκB signaling was attenuated with OLE treatment through a reduction in p65 translocation to the nucleus and restoration of normal protein levels of IκB-α in the cytosol [58]. OLE increased mRNA of M2 polarization markers ARG-1 and CD206 and the transcription factor PPARγ (Table 10) [58].

Mirsanei et al. [59] stimulated RAW 264.7 cells with LPS in the presence of increasing concentrations of OLE, which led to a decrease in pseudopod length and a shift in cell shape, becoming more spherical in nature [59]. OLE treatment led to the downregulation of mRNA levels of pro-inflammatory M1 factors IL-12, IFN-γ, iNOS, and TNFα [59]. OLE induced a shift to an M2 phenotype through upregulation of IL-10 and TGF-β mRNA levels. LPS stimulation led to increases in NO production, while incubation with OLE attenuated such effects [59]. When in contact with a pathogen, cell debris, or a foreign molecule, macrophages have the ability to phagocytose and lyse this molecule as part of the innate immune system [59]. When treated with OLE, this ability to phagocytose was greatly reduced, as demonstrated by the quantification of yeast phagocytosis (Table 10) [59].

## 6. Conclusions

In summary, the in vitro findings on insulin target tissues, muscle, and adipose demonstrated the ability of OLE to increase GLUT4 plasma membrane levels and phosphorylation/activation of AMPK, resulting in increased glucose uptake. Furthermore, OLE was able to attenuate FFA-induced insulin resistance while decreasing the levels of ROS. In adipocytes, OLE led to a reduction in lipid accumulation and mRNA levels of multiple pro-adipogenic genes, indicating anti-adipogenic properties. Furthermore, OLE attenuated pro-inflammatory cytokine levels, demonstrating anti-inflammatory properties. In hepatocytes, OLE decreased lipid accumulation through a reduction in adipogenesis-related genes, indicating an ability to combat hepatic steatosis. Moreover, oleuropein increased hepatic mitochondrial activity.

Similarly to muscle cells, OLE was able to abrogate increased levels of ROS in beta pancreatic cells, particularly through increases in levels of anti-oxidant enzymes. OLE markedly increased insulin secretion from beta pancreatic cells even in the presence of elevated pro-inflammatory mediators.

The findings of the in vivo studies showed the ability of OLE to prevent HFD- induced bodyweight gain and lipid accumulation in insulin target tissues. OLE administration led to decreases in plasma levels of TC, TG, and LDL-C and an increase in HDL-C. Furthermore, OLE led to reductions in blood glucose levels and increased insulin sensitivity, demonstrating anti-diabetic properties. OLE increased UCP-1 levels in adipose tissue, suggesting increased thermogenesis. In liver and adipose tissue, OLE led to downregulation of multiple pro-adipogenic genes and pro-inflammatory markers, indicating anti-adipogenic and anti-inflammatory properties. Additionally, OLE promoted the activity of certain anti-oxidant enzymes while encouraging a healthy shift in the gut microbiome.

While there have been a number of in vitro and in vivo studies that have indicated OLE to have anti-diabetic properties, there has been a lack of clinical/human studies. In the existing in vivo animal studies, different doses of OLE were used, and it is not possible to know the effective dose in humans unless clinical studies are performed. Clinical studies are also required to determine potential toxicity and examine OLE’s anti-diabetic effects in humans.

In addition, there are no studies comparing the effects of OLE with the effects of established diabetes medications such as metformin. Such comparisons are necessary to establish OLE as an anti-diabetes agent.

Further research to determine the effective doses and anti-diabetic potential of OLE in humans is needed. Long term clinical studies are required to examine potential toxicity and side effects. At this point, without the scientific evidence from clinical trials, no recommendations should be made relating to its use in humans.

## Figures and Tables

**Table 1:** Effects of Oleuropein on Skeletal Muscle Cells (in vitro).

Source	Cell Type	Treatment	Findings
[22]	C2C12 myotubes n = 3	OLE 200 µM or 400 µM for 24 h H_2_O_2_ treatment for 30 min at 400 µM	↑ Glucose consumption ↑ Insulin sensitivity ↓ H_2_O_2_-induced ROS ↑ p-AMPK, p-ACC, and p-ERK protein levels ↑ p-ERK protein levels
[23]	C2C12 myotubes n = 3	OLE 1 µM, 10 µM, or 100 µM for 30 min to 24 h 250 µM palmitic acid for 24 h	↑ Glucose uptake ↑ GLUT4 mRNA levels ↑ p-AMPK protein levels
[24]	Male chicks (Ross strain, *Gallus gallus domesticus*) n = 4–6	OLE Oral admin. 5 mg/kgbw/day 15 days	↑ avUCP, PGC1-α, TFAM, NRF1, ATP5a1, and SIRT1 mRNA levels ↑ Cytochrome C oxidase activity ↓ Mitochondrial superoxide activity (ROS production)
[25]	Soleus Muscle (isolated from male Sprague-Dawley rats) n = 6	OLE 1.5 mM	↑ p-AMPK and GLUT4 ↑ Glucose uptake Inhibition of OLE-induced effects with Compound C
[26]	Soleus muscle (isolated from male Sprague–Dawley rats) n = 6	OLE 1.5 mM 12 h (1.5 mM, 12 h)	Glucose uptake p-AMPK, AS160, and GLUT4 protein levels ↓ Improvements during exposure to Compound C

Table Legend: ↑ = increase ↓ = decrease → = no effect p- = phosphorylated. Only findings reported as statistically significant in the original manuscripts are included in this table.

**Table 2:** Effects of Oleuropein on Adipocytes (in vitro).

Source	Cell Type	Treatment	Findings
[27]	3T3-L1 Pre-adipocytes n = 3	OLE 0–400 µmol/L for 0–8 days	↓ Glucose uptake, total lipid, and TG levels ↓ GDPH protein levels ↓ PPARγ, C/EBPα, CD36, and GLUT4 mRNA levels ↓ SREBP-1c and FASN mRNA levels ↑ G0/G1 and S population of cells
[28]	3T3-L1 Pre-adipocytes n = 3	OLE 1, 10, 200 and 400 µM for 11 days	↓ PPARγ, C/EBPα, SREBP-1c, and FAS mRNA levels
[29]	3T3-L1 Pre-adipocytes n = 3	OLE 0.1, 1, 10, 50, and 100 µM for 10 days SFRP2 100 nM	↓ Lipid accumulation ↓ Adipogenesis (SRFP2/galnon-induced) ↓ PPARγ, C/EBPα, FASN, and LPL mRNA levels
[30]	3T3-L1 Adipocytes n = 3	OLE 100 and 300 µM for 24 h	↑ Insulin stimulated p-IRS protein levels (100 µM)

Table Legend: ↑ = increase ↓ = decrease → = no effect p- = phosphorylated. Only findings reported as statistically significant in the original manuscripts are included in this table.

**Table 3:** Effects of Oleuropein on Hepatocytes (in vitro).

Source	Cell Type	Treatment	Findings
[31]	HepG2 and FL83B hepatocytes n = 3	OLE 10 and 50µM for 24 h 0.5 mmol/L 2:1 oleic acid–palmitic acid	↓ Lipid accumulation/droplet size → TIP47 and ADRP mRNA levels ↓ FFA-induced p-ERK protein levels → p-JNK and p-Akt protein levels
[32]	FaO cells n = 3	OLE 50 µg/mL for 24 h 0.75 mM 2:1 oleate: palmitate	↓ TG accumulation ↓ Lipid peroxidation/oxidative stress
[33]	HepG2 hepatocytes n = 6	OLE 10 µM for 2 and 24 h	OLE acts as a PPARα ligand ↑ PPARα mRNA and protein levels ↑ ACOX1, CYP4A14, Lipin 1, and ACOT4 mRNA levels
[34]	HepG2 hepatocytes n = 5	OLE 10, 25, 50, 100 and 200 µM for 24 h Steatosis model 0.5 mM (PA/OA)	↓ Lipid accumulation (50, 100, and 200 µM)
[35]	AML-12 cells n = 3	OLE 10, 40, 80, 160 µM for 24 h 0.25 µM oleic acid for 24 h	↓ Lipid droplet formation, TG accumulation ↓ ACC, SREBP-1c, and FASN mRNA levels ↑ PGC-1α, LKB1, Nrf2, PGC-1α, and Tfam mRNA levels ↑ LKB1 binding at PGC-1α promoter

Table Legend: ↑ = increase ↓ = decrease → = no effect p- = phosphorylated. Only findings reported as statistically significant in the original manuscripts are included in this table.

**Table 4:** Effects of Oleuropein on Pancreatic Beta Cells (in vitro).

Source	Cell Type	Treatment	Findings
[36]	INS-1 insulinoma cells n = 3	OLE 0.0001–0.1 mmol/L for 24-h 0.035 mM H_2_O_2_ for 45 min	Improved cell viability ↓ H_2_O_2_ induced necrotic cell death ↑ Number of living cells ↓ H_2_O_2_ induced ROS production
[37]	INS-1 insulinoma cells n = 7	OLE 0.0001–0.1 mM for 24-h 0.15 ng/mL IL-1β, 1 ng/mL IFN-*γ*, 1 ng/mL TNFα for 6 h	↑ Cell viability in cytokine presence ↓ ROS, ↑ SOD ↑ Insulin secretion in cytokine presence
[38]	INS-1 insulinoma cells n = 3	OLE 10 µM for 1 or 24 h	↑ Insulin content ↓ Glucose stimulated insulin release
[39]	INS-1 insulinoma cells n = 3	OLE 0.1, 1 and 10 µM, 30 min pre-treatment 48 h TCDD	↓ COX-1 and PGE_2_ mRNA levels (0.1, 1, and 10 µM) ↓ iPLA_2_ and PLA_2_G10 (10 µM), TNF-α, and p-JNK1 mRNA levels ↑ p-Akt mRNA levels (0.1 µM) ↓ ROS production (0.1, 1, and 10 µM) ↑ GLUT2 mRNA levels

Table Legend: ↑ = increase ↓ = decrease → = no effect p- = phosphorylated. Only findings reported as statistically significant in the original manuscripts are included in this table.

**Table 5:** Effects of Oleuropein on HFD-induced Diabetes Animal Models (in vivo).

Source	Animal Type	Treatment	Findings
[40]	Male Sprague–Dawley rats n = 6	OLE 1, 2 or 4 mg/kg or 0.1, 0.2, or 0.4% dietary supplementation for 28 days HFD (30% shortening)	↓ Body weight/bodyweight gain ↓ Weight of epididymal fat pad ↓ Perirenal adipose tissue weights ↓ Plasma TG, TC, FFA, and leptin levels ↑ IBAT UCP-1 protein levels ↑ Urine levels of norepinephrine and epinephrine
		OLE 10–50 mmol/L for 10 min	↑ Plasma levels of norepinephrine and epinephrine
[41]	Male Wistar rats n = 10	3 mg/kg (of body weight) for 16 weeks HCD 1% cholesterol and 0.25% bile salts	↓ Liver/bodyweight ratio ↓ Plasma TC, TG, and LDL-C levels, ↑ Plasma HDL-C levels ↑ SOD and CAT activity, ↑ Antioxidant activities (TEAC assay) ↓ Lipid peroxidation Prevented cardiac muscle hypertrophy, aortic wall lesions, and hepatic steatosis
[42]	Male C57BL/6N mice n = 8	OLE dietary supp. 0.03% (*w*/*w*) for 10 weeks	↓ Oxidative stress/pro-inflammatory related hepatic genes ↓ Lipid peroxidation product detoxification related hepatic genes ↓ Hepatic mRNA levels of fatty acid uptake/transport genes
[43]	Male C57BL/6N mice n = 8	OLE admin. ad libitum 0.03% (*w*/*w*) for 10 weeks	↓ Bodyweight gain and liver weight ↓ Plasma AST and ALT ↓ Plasma and liver FFA TC, and TG ↓ Liver LXR, PPARγ2, LPL, aP2, Cyc-D, E2F1, CTSS, SFRP5, DKK2 mRNA levels ↓ Liver p-ERK protein level ↑ Liver β-catenin protein level ↓ Liver TLR4, TLR2, MyD88, TNF-a, IL1β, IL-6, IFNβ, FAS, & TRAIL
[29]	Male C57BL/6N mice n = 8	OLE 0.03% (*w*/*w*) for 10 weeks	↓ Bodyweight gain ↓ Total visceral fat pad weight, ↓ Adipocyte size ↓ Plasma TG, TC, and FFA levels ↓ Epididymal SFRP2, DKK2 mRNA levels, ↑ Wnt10b mRNA levels ↓ Galanin, Gal1R1, GalR2, PKCδ, RAS mRNA levels ↑ β-catenin protein levels, ↓ p-ERK protein levels ↓ PPAR*γ*, C/EBPα, LPL, FASN, aP2 mRNA levels
[44]	Male C57BL/6JOlaHsd mice n = 5	OLE 20 mg/kg daily for 15 weeks Peracetylated derivative 20 mg/kg daily for 15 weeks (in drinking water) CAF diet (26% fat, 52% carbs)	↓ HFD-associated weight gain ↓ Blood glucose, insulin levels ↓ HOMA-IR level ↓ Liver weight % steatosis ↓ Plasma LDL-C, ALT, AST levels ↓ Leptin levels
[45]	Male Wistar rats n = 5	oleuropein supplementation (50 mg/kg), via gavage for 8 weeks HCD 10% cholesterol, 5% fructose, and 0.1% bile salts	↓ HFD-induced body weight gain ↓ Liver weight, ↓ Hepatic TG content ↓ Adipose tissue mass/adipocyte size ↓ Serum LDL-C and TG, ↑ Serum HDL-C ↑ Adiponectin mRNA level ↓ PPARγ, C/EBPα, and FASN protein levels, ↑ p-AMPK protein levels
[46]	Male albino rats n = 10	OLE 10 mg/kg/day 12 Weeks	↓ Total body weight ↓ Total Cholesterol and LDL cholesterol ↑ HDL Cholesterol
[23]	Male C57BL/6J mice n = 6	0.38 g (0.038% of diet) for 12 weeks HFD (200 g lard, 300 g sucrose)	→ White/brown adipose tissue weight ↓ Fasting blood glucose level ↑ Insulin sensitivity → Fasting blood insulin levels and AUC → Blood non-esterfied fatty acid levels ↑ GLUT4 levels (IHC)
[34]	Male C57BL/6J mice n = 8	OLE 0.03% *w*/*w* by oral gavage For 8 weeks	↓ Heart weight ↓ Serum AST, ALT, and TC ↑ Serum HDL-C ↓ Serum IL-1α, G-CSF ↑ Hepatic CAT, SIRT1/3 protein levels ↓ Hepatic MDA levels
	Female C57BL/6J mice n = 8	OLE 0.03% *w*/*w* by oral gavage For 8 weeks	↓ Heart weight ↓ Serum AST, ALT, and TC ↑ Serum HDL-C ↓ Serum IL-2, G-CSF ↑ Hepatic SOD, CAT, and SIRT1/3 protein levels ↓ Hepatic MDA levels
[30]	Male rats n = 8	50 mg/kg (oral gavage) for 8 weeks HFD 10% sheep fat, 5% fructose, and 0.1% bile salts)	→ Bodyweight gain ↓ Blood glucose level ↓ Blood insulin level, HOMA-IR, and AUC ↓ Serum TG, LDL-C, TC levels ↑ Serum HDL-C level ↑ Pancreatic IRS1 level (IHC) ↑ Liver and WAT p-Akt, IRS1, and GLUT4 protein levels ↓ Liver and WAT TNFα protein level
[35]	Male C57BL/6J mice n = 6–8	OLE 0.6% ad libitum for 8 weeks HFD (45% calories from fat)	↓ Bodyweight gain and liver weight ↓ Hepatocyte swelling and ballooning ↓ Serum ALT and AST levels ↓ Serum TC, TG, and LDL-C levels ↓ Hepatic MCP-1, CD68, SREBP-1c, ACC, and FASN mRNA levels ↑ Hepatic PGC-1α, LKB-1, Tfam, Nrf2, UCP2, and mtDNA mRNA levels
[47]	Male C57BL/6N Mice n = 10	OLE Dietary supp. 0.03% (*w*/*w*) for 16 weeks	↓ Bodyweight gain and epididymal weight ↓ Hepatic lipid accumulation ↓ Serum AST, ALT, TC, TG, LDL-C ↓ Hepatic MDA, GPx, SOD, and CAT protein levels ↑ Hepatic nicotinamide, tauroursodeoxycholic acid, taurine, and docosahexaenoic acid ↑ PPARα, FGF21, CPT1α, and ACOX1 protein levels ↓ FAS, SREBP-1c, and SCD1 protein levels ↓ IL-6, TNFα, MCP-1 hepatic mRNA levels
[48]	Male and female C57BL/6N Mice n = 13	OLE 1.8 mg/kg ad libitum for 8 weeks	↓ Steatosis and fibrosis score ↓ Serum LPS, Zonulin, sP-selectin level ↓ Serum HDL3, TNFα, IFN*γ* level ↓ Hepatocyte LPS localization, number of TLR4+ macrophages, number of CD42b+ platelets (IHC) ↓ intestinal goblet cell score and crypt length ↓ Enterocyte LPS localization, occludin, number of TLR4+ macrophages (IHC)

Table Legend: ↑ = increase ↓ = decrease → = no effect p- = phosphorylated. Only findings reported as statistically significant in the original manuscript were included in this table.

**Table 6:** Effects of Oleuropein on Alloxan-induced Diabetes Animal Models (in vivo).

Source	Animal Type	Treatment	Findings
[49]	Rabbits n = 8	20 mg/kg b.w. for 16 weeks	↓ Blood glucose level ↓ Plasma and erythrocyte MDA and SOD levels ↑ Plasma and erythrocyte levels of GPx, GRx, CAT, GSH, α-tocopherol, β-carotene, and ascorbic acid ↓ Plasma erythrocyte abundance
[50]	Male Wistar rats n = 10	16 mg/kg b.w. oleuropein for 4 weeks	↓ Blood glucose level ↑ Hepatic glycogen level ↓ Plasma TC ↑ CAT activity, hepatic SOD protein levels ↑ Antioxidant activity (TEAC) ↓ Hepatic TBARS levels ↓ Hepatic steatosis

Table Legend: ↑ = increase ↓ = decrease → = no effect p- = phosphorylated. Only findings reported as statistically significant in the original manuscripts are included in this table.

**Table 7:** Effects of Oleuropein on Streptozocin-induced Diabetes Animal Models (in vivo).

Source	Animal Type	Treatment	Findings
[51]	Male Sprague–Dawley rats n = 8	20, 40 or 60 mg/kg/day for 4 weeks STZ (65 mg/kg)	↓ SBP ↓ FBG levels ↓ Serum LDL-C and TG levels ↓ Serum TC levels (40 and 60 mg/kg/day) ↑ HDL-C levels (60 mg/kg/day) Restored alloxan induced ↓ insulin levels
[52]	Male Sprague–Dawley rats, STZ-induced diabetes n = N/A	OLE oral admin. 5 mg/kgbw/day 15 days	↓ Seurm ALT, AST, alkaline phosphatase, and bilirubin serum protein content ↓ Seurm TC, TG, LDL-C, and leptin ↑ HDL-C, and adiponectin ↓ TNF-α and COX-2 mRNA levels

Table Legend: ↑ = increase ↓ = decrease → = no effect p- = phosphorylated. Only findings reported as statistically significant in the original manuscripts are included in this table.

**Table 8:** Effects Oleuropein on Diabetes Phenotypical Animal Models (in vivo).

Source	Animal Type	Treatment	Findings
[53]	Male wild-type BKS-DB (m/m) mice and male BKS-Lepr*^em2Cd479^*/Gpt (*db*/*db*) mice n = 8	OLE 200 mg/kg for a 4 h or 15-week period	→ Body weight, ↓ epididymal fat mass → Insulin sensitivity ↓ Fasting blood glucose levels ↑ Pancreatic islet area ↓ Hepatic lipid accumulation ↑ Liver p-Akt protein ↓ Fbp1 mRNA levels ↓ Pepck and G6pase mRNA levels Altered gut microbiome
[54]	Male BKS–Leprem2Cd479/Gpt (db/db) mice n = 8	OLE 200 mg/kg bw daily oral admin. for 15 weeks	↓ Glomerular mesangial matrix expansion (PAS) ↓ Renal fibrosis ↓ F4/80, cleaved caspase-3, BAX protein levels (renal and cardiac tissue) ↑ Bcl-2 protein levels (cardiac and renal tissue) ↓ NADPH oxidase 4 mRNA level (renal tissue) ↑ cGMP-PKG/Gap junction signaling genes (renal tissue) ↓ p53 and cell senescence signaling genes (cardiac tissue)

Table Legend: ↑ = increase ↓ = decrease → = no effect p- = phosphorylated. Only findings reported as statistically significant in the original manuscript were included in this table.

**Table 9:** Effects of Oleuropein in Humans (clinical studies).

Source	Animal Type	Treatment	Findings
[55]	Human (males) (35–55 years old) (BMI 25–30 kg/m^2^) n = 46	OLE Orally 62 mg capsule four times daily for a 12-week period	→ Body composition ↑ Energy intake (kcal) ↑Insulin sensitivity (IGTT), and glucose tolerance (OGTT) ↑ IL-6 mRNA levels → IL-8, TNFα, and CRP mRNA levels ↑ Plasma IGFBP-1/2

Table Legend: ↑ = increase ↓ = decrease → = no effect p- = phosphorylated. Only findings reported as statistically significant in the original manuscripts are included in this table.

**Table 10:** Effects of Oleuropein on Macrophages in culture (in vitro).

Source	Cell Type	Treatment	Findings
[56]	RAW 264.7 cells n = 3	OLE 100, 200 and 300 µM for 1 and 24 h	↓ NO production and iNOS mRNA levels ↓ COX-2, L-1β, and IL-6 mRNA levels ↓ p-IκB-α protein content, NFκBp65 nuclear translocation ↓ p-ERK, JNK, and AP-1 protein levels
[57]	Murine peritoneal macrophages n = 4	OLE 25 and 50 µM for 18 h	↓ ROS production ↓ NO production and iNOS protein levels ↓ IL-1β, IL-6, PGE_2_ mRNA levels ↓ JNK, ERK, p-STAT3 and p38 protein levels
[58]	RAW 264.7 cells n = 3	OLE 0.1 and 0.2 mg/mL for 24 h	↓ ROS (palmitate-induced) ↓ % of iNOS+ cells and iNOS protein levels ↑ NRF2 nuclear translocation ↑ PPARγ, GCLC, GCLM, and HMOX-1 mRNA levels ↓ KEAP1 protein level in cytosol ↓ TNFα, IL-6, IL-1β, COX-2 mRNA levels ↑ Cytosolic IκB-α, ↓ NFκBp65 levels in nuclei ↑ ARG-1+ and CD206+ cells ↑ ARG-1ARG-1, IL-10, and CD206 mRNA levels
[59]	RAW 264.7 cells n = 3	OLE 50, 100 and 200 µg/mL for 24 h	↓ Pseudopod length, altered cell shape ↓ IL-12, IFN-γ a, and TNFα mRNA levels ↓ iNOS mRNA levels and NO production ↑ IL-10 and TGF-β mRNA levels ↓ Phagocytotic activity

Table Legend: ↑ = increase ↓ = decrease → = no effect p- = phosphorylated. Only findings reported as statistically significant in the original manuscripts are included in this table.
